# Developing and validating a measurement tool to self-report perceived barriers in substance use treatment: the substance use treatment barriers questionnaire (SUTBQ)

**DOI:** 10.1186/s13011-021-00419-1

**Published:** 2021-11-07

**Authors:** Hamid Tavakoli Ghouchani, Hossein Lashkardoost, Hassan Saadati, Seyed Kaveh Hojjat, Faezeh Kaviyani, Kazem Razaghi, Dordane Asghari, Nazanin Gholizadeh

**Affiliations:** 1 Addiction and Behavioral Sciences Research Center & Department of Health Education and Promotion, School of Health, North Khorasan University of Medical Sciences, Bojnurd, Iran; 2 Addiction and Behavioral Sciences Research Center & Department of Epidemiology and Biostatistics, School of Health, North Khorasan University of Medical Sciences, Bojnurd, Iran; 3 Addiction & Behavioral Sciences Research Center, Department of Internal Medicine, School of Medicine, North Khorasan University of Medical Sciences, Bojnurd, Iran; 4 Division of sleep medicine, Psychiatry and Behavioral sciences research center, Mashhad university of medical sciences, Mashhad, Iran; 5 Addiction and Behavioral Sciences Research Center, North Khorasan University of Medical Sciences, Bojnurd, Iran; 6 PhD, Lecturer, School of Nursing, Midwifery and Paramedicine NSW/ACT, Australian Catholic University, Sydney, Australia; 7 Measurement & Measurement Field (Psychometric Field), Addiction & Behavioral Sciences Research Center (Researcher), Bojnurd, Iran; 8 General Psychology, Addiction & Behavioral Sciences Research Center, Bojnurd, Iran

**Keywords:** Questionnaires, Perceived barriers, Substance use, Validity, Factor structure

## Abstract

**Background:**

Substance using often cause a wide range of social, health, and psychological problems. This study aimed to develop and validate a questionnaire of barriers of treatment in substance users.

**Methods:**

In this cross-sectional study, the initial questionnaire was designed based on the evaluation of previous studies. The preliminary tool including 35 Likert-scaled items. After assuring the face validity of the questionnaire, 13 experts’ opinions were obtained for assessing or improving the content validity. The reliability was investigated by internal consistency methods using Cronbach’s alpha. For measuring the structural validity, the exploratory factor analysis was performed to determine the dimensionality of the questionnaire using principal components extraction and Varimax rotation.

**Results:**

The preliminary questionnaire consisted of 35 items. After completing the face validity and summarizing the experts’ suggestions, 8 items were removed. By calculating the content validity ratio and coefficient, 11 questions were deleted. The internal consistency was calculated to be 0.84 using Cronbach’s alpha. In the last stage and according to the results of the factor analysis, three factors fear of or unawareness of treatment, doubt or inefficiency, and social stigma were identified from the 10-items questionnaire, which explained 67.34% of the total variance.

**Conclusion:**

Considering the necessity of using a validated tool for planning and evaluating effective interventions on people who use substance is inevitable. The Substance use Treatment Barriers Questionnaire is designed with 10 items and 3 dimensions, which has appropriate validity and reliability and can be used to determine the obstacles for treatment or factors that lead to discontinuing treatment.

## Introduction

Substance use is an old issue in today’s world [1]. According to WHO, in eastern Mediterranean regions, the prevalence of substance use disorders is estimated about 3.5% [2]. In Iran, due to being located close to a major pathway for the trade of narcotics, illegal substances particularly opioids are widely accessible, and thereby drug addiction is a major concern [3, 4]. According to some studies opium consumption enhance the risk of some of cancers for example esophageal cancer [5, 6]. In addition, substance using has many consequences affecting person with a substance using disorder, and their families [7].

According to reports of the Iran Drug Control Headquarters, there are 2 million (2.5% of the population) substance-dependent people in Iran [8]. Also It is estimated that the prevalence experience of drug using among Iranian adults was 11.9% [9].

There are comprehensive programs to treat and control of drug use in Iran, which included activities focused on drug supply reduction, drug demand reduction and harm reduction [10]. Considering people who use drugs as patients, there are a number of preventive and treatment programs in progress in primary health care systems in Iran [11]. Treatment of addiction, due to its particular nature, requires appropriate combination of approaches [12, 13]. In many cases, persons who treat for drug using, do not enter to any rehabilitation program or leave such programs in early stages of treatment [14]. Many of these individuals either think they do not have any problems [15] or prefer to manage the issue on their own, and thereby do not seek professional help [13, 16]. Obstacles of successful treatment of substance dependence has been researched in different parts of the world [17, 18]. Raising awareness and understanding about the legal, medical, and social aspects of substance use can help reduces obstacles to treatment and prevent further consequences [19].

According to some studies, some of the obstacles for starting the substance use treatment and success of treatment processes include low level of patients’ trust for the treatment services and lack of access to professional medical teams [15, 20]. Several studies have shown other obstacles including personal and family issues, lack of medical insurance, financial issues [12, 21], side effects of treatment [16], fear of being deprived from substances [22], inability to proceed treatment processes [23], a positive attitude towards substances [24], lack of preparedness for the process [23], lack of awareness about the process of treatment [25], and unwillingness to notify or to be known by others [23]. Other studies have mentioned personal factors [17] such as lack of self-efficacy, failure to acknowledge one’s situation as a health disorder, absence of enabling and empowering factors, cultural values and gender related factors among the hindrances for taking action to abandon substance use [26–28].

While most existing studies have investigated obstacles for treatment or factors that lead to discontinuing treatment, less attention has been given to obstacles for starting treatment. Also, previous studies have addressed only partially personal/family, cultural, or social aspects of initiating treatment. However, in the present study, through a questionnaire developed based on a review study of obstacles for initiating treatment and discontinuing treatment, a broad and comprehensive approach was adopted to explore such obstacles. Finally, by adopting a solution-based view, approaches are suggested for removing those obstacles. There is limited questionnaires relating to treatment barriers thus the study aims to develop a timely scale to be used to determine barriers to treatment programs among people of who use drugs in Iran - as the older ones may not be able to capture the changing treatment challenges faced by people of who use drugs.

## Methods

A psychometric study was conducted to develop a self-reporting tool for assessing Perceived Barriers in Substance Use Treatment: The Substance Use Treatment Barriers Questionnaire (SUTBQ). It was prepared in Persian and administered on patients referring to Methadone maintenance treatment (MMT) clinics and undergoing maintenance treatment with methadone in Bojnurd of Iran in 2019. Sampling was conducted in cluster sampling form as the city was divided into seven districts, and from each district, addiction rehabilitation clinics were identified. Being mindful of the more crowded districts, overall, 10 clinics were randomly selected. Upon obtaining the necessary approvals, 40 individuals were selected from each clinic. Ultimately, the study involved an overall 384 individuals. Inclusion criteria were those who used drugs; those who were in the first month of treatment; willingness to cooperate and had no previous withdrawal history. Exclusion criteria also included inability to respond and problems with memory (forgetfulness-dementia). The potential participants were explained the aim, importance, benefits, and potential risks of the study and told that they could withdraw from the study at any point. Then they decided if they would like to participate in the research or not. The participants were assessed and interviewed upon entering the clinics for initiating treatment processes. Informed assent and consent were obtained from participants. The study was conducted with approval from North Khorasan University’ Institutional Review Board and Ethical Committee. After developing the preliminary version of the questionnaire, internal consistency were used to assess reliability. Also in order to ensure validity of the tool, face validity, content validity, and structural validity were applied.

### Questionnaire design

**Fig. 1:**
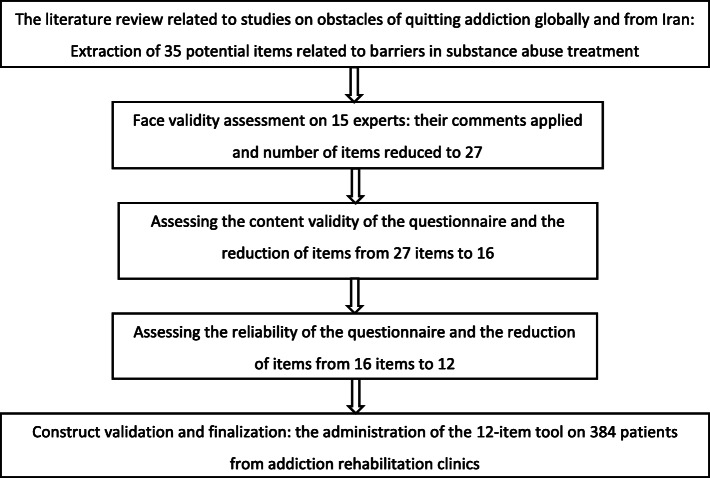
Process used for developing and assessment of the questionnaire

To design the questionnaire, a literature review was initially conducted by the research team in association with studies on obstacles of quitting addiction. A preliminary tool including 35 Likert-scaled items was developed after extracting the initial information through focus group discussions by a team of experts (including psychologist, general practitioner, psychiatrist, epidemiologist) and stakeholders (who involved in the treatment of drug addiction in the clinics). This tool was evaluated for both content and face validity through view of the expert panel. Details of the steps for preparing and evaluating the questionnaire are provided in Fig. 1.

### Face validity

The face validity is the degree of the respondents judgment to which the appearance of the tool is suitable for collecting the information [29]. In order to evaluate the face validity, the preliminary questionnaire was sent out to 15 experts in field of psychiatry, psychology, epidemiology, and health promotion. Expert opinions were collected during a period of 30 days.

### Content validity

Content validity is defined as “the degree to which items in an instrument reflect the content universe to which the instrument will be generalized, in other words the purpose of content validity is to ensure the ability of the tool to measure the concept that it claims to measure [30]. To assess the content validity of the Persian SUTBQ, all the items were sent out to 15 experts in various fields and they were asked to give their comments for each item in three aspects of relevance, clarity and necessity. They were asked to respond regarding each of the aspects of item assessment through a Likert scale of four choices including; “I totally agree”; “I agree” “I disagree”; and “I totally disagree”. In case an expert disagreed with each item, he/she was given an opportunity in order to give explanations or improvement suggestions. Twelve experts agreed to contribute who were provided face-to-face or by e-mail with a content validity assessment package. These experts were from a range of expertise in various field of psychiatry, psychology, epidemiology, and health promotion. After assessments, the Content Validity Ratio (CVR) and Content Validity Index (CVI) were calculated.

### Reliability

After assessing the validity and preparing the 16-item questionnaire, the reliability of the questionnaire was assessed using internal consistency (Cronbach’s alpha) method. Cronbach’s alpha coefficient of more than 0.7 were the cutoff value to confirm the internal consistency of each subscale.

### Construct validity

For measuring the structural validity at this stage, after evaluating the validity and reliability of the questionnaire and finalizing it, exploratory factor analysis was performed to determine the dimensionality of the questionnaire using principal components extraction and Varimax rotation [31, 32]. The sample size was determined by scientific references in exploratory factor analysis and was estimated based on the number of items in the questionnaire multiplied by 6–10 as recommend [33]. For this stage of the study, 384 participants entered the study by cluster sampling. Factor loading values of 0.5 or higher, were considered acceptable, and showed that there was an important relationship between items and factors. In order to evaluate sampling adequacy to perform a satisfactory factor analysis, KMO Measure of Sampling Adequacy and Bartlett test was high values of KMO (more than 0.7) generally indicated that a factor analysis may be useful with the data. The criteria used to determine the subscales (factors) were minimum Eigenvalues > 1.00 (Kaiser Criterion). Ethical approval for this study was obtained from Research Ethics Committee in North Khorasan University of Medical Sciences (IR.NKUMS.REC.1397.020).

### Statistical analyses

All exploratory factor analysis steps were performed using SPSS V.22 software.

## Results

The majority of the participants were male (77.3%). The average age of the studied group was (47.75 ± 13.3 years). The majority of the studied individuals were married (81.7%), and more than half (64.6%) had studied up to junior high school. The average age for the first time uses of drugs was (29.95 ± 10.73 years) and the youngest and the oldest participants were 20 and 87 years old, respectively. The shortest and the longest duration of addiction were 1 year and 61 years, respectively. In total, the average duration of drug use was (17.47 ± 11.47 years), and 293 people (80.7%) did not report any particular previous health issues. More than half of the participants (63.2%) had attempted to quit for the first time. 43.5% of the participants reported that there were one or more drug users in their own family.

The preliminary questionnaire consisted of 35 items prepared by the core expert panel. In the preliminary questionnaire, 8 questions were removed through the face validity assessment process and the questionnaire with 27 questions was finalized at this stage. Similarity, ambiguity, and controversy with the purpose of the study, were the reasons for removing items by experts. In content validity assessment, CVR and CVI results indicated that all of the questions, except for 11 questions, had a score higher than 0.78 and therefore were recognized necessary and relevant. Therefore, the 27-item questionnaire was reduced to a 16-item questionnaire to be assessed for reliability. In the assessment of reliability by Cronbach’s alpha, four items were removed. The internal consistency was calculated to be 0.803 using Cronbach’s alpha for SUTBQ. Finally, by removing 4 items due to low reliability, the tool was reduced to a questionnaire with 12 items to be entered into factor analysis step.

**Fig. 2:**
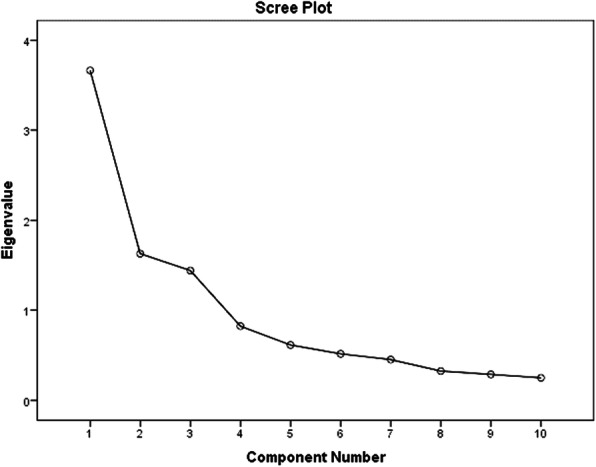
Scree plot for factor components of the questionnaire

Construct validity was determined as following: In the first step, Kaiser Meyer-Olkin (KMO = 0.745) and Bartlett’s Test (*P* < 0.001, Chi-Square = 1450.639, df = 45) showed the adequacy of the sample size. Principal component analysis with Varimax rotation identified three factors (Eigenvalues > 1.0, factor loading cut off ≥0.5) which explained 67.34% of the variance in the data. The number of factors was also confirmed by the scree plot (Figure 2).

Through an iterative process for exploratory factor analysis, the uniqueness of each item was examined and any item with a uniqueness score higher than 0.7 was removed after examining and comparing with other questions, which reduced the number of questions to 10 questions.

In general, three factors with eigenvalue > 1 were extracted. These factors could explain 67.34% of the total scale variance. These three factors of the SUTBQ, which had subscales, named and presented in Tables 1 and 2.

**Table 1:** Three factors comprised the subscales of the SUTBQ

Subscales	Perceived Barriers	Cronbach’s alpha
Subscale 1	Fear of or Unawareness of treatment	0.771
Subscale 2	doubt or inefficiency	0.743
Subscale 3	social stigma	0.825
Total		0.803

**Table 2:** The related factors and factor loads using principal component analysis method with Varimax rotation

Questions barriers of substance abuse treatment (SUTBQ)	Fear of or Unawareness of treatment	doubt or inefficiency	social stigma
Because I was unaware of social and physical consequences of addiction, I did not try to quit.	.696		
Because I was afraid my psychological situation would worsen, I did not try to quit.	.732		
Because I had not found any effective methods for treatment, I did not try to quit.	.798		
Because of unawareness of the effects of alternative medicines and the stages of treatment, I did not try to quit.	.695		
Because I doubted, I would be able to continue the treatment process, I did not try to quit.		.554	
Because there were many addicts around me, I did not have the right motivation to quit.		.755	
Due to lack of family support, I did not try to quit.		.685	
Due to the feeling of joy and pleasure from the drugs, I did not try to quit.		.822	
Due to fear of disclosure of my privacy from the clinic, I did not try to quit.			.847
Because of labeling and social stigma for addiction, I did not try to quit.			.903

The internal consistency statistics of the final SUTBQ showed that after exploratory factor analysis were satisfactory for the whole scale and all its subscales. The Cronbach’s alpha for SUTBQ whole scale was 0.803. The lowest Cronbach’s alpha was calculated to be 0.743 belonging to “doubt or inefficiency” subscale.

## Discussion

There are a few questionnaires relating to treatment barriers, but unfortunately their use seems inappropriate among a vast population of people of who use drugs. Results of the present study show that Fear of or Inefficiency of treatment, Doubt or Unawareness of treatment, and Social Stigma are some of the obstacles for quitting substance use. Doubt or Inefficiency of treatment are among the obstacles for quitting drug use. This feeling can rise from external and social factors such as lack of clear instructions or from cultural obstacles such as lack of patient’s adequate awareness in terms of the treatment. This can also stem from the patient’s lack of self-efficacy and lack of enabling and empowering factors in the person who uses substance [26–28], which largely are consistent with the results of the present study.

A study by Pennay and Lee (2009), through a survey of providers of medical services for substance use, reported that the most common obstacles or low rate of success in quitting methamphetamine were low budget and resources, limited access to services, lack of awareness about treatment options, social stigma, low organizational coordination, inefficient management of cases and follow up [34]. Pennay and Lee (2009) stated that lack of clear instructions and a known protocol result in approaches with short term effects. This, in turn, causes that due to lack of trust in the treatments provided by medical services, individuals’ decision to quit depend on personal decisions [34] which is indicative of a sense of doubt and distrust in treatment. This is also observed in the findings of the current study.

Manuel et al. (2017) found that lack of family support can be an individual-family obstacle which can cause doubt and inefficiency in considering quitting. As it is shown in the current study, sense of inefficiency can be a reason for lack of trust in starting treatment [18]. In this regard, having informal support networks such as family, friends, and people can reduce participants’ worries. Ashford et al. (2018) reported sense of concern about support services including family support and post-treatment support services such as consultation, follow up, and specialized sessions, among the obstacles for quitting addiction [35]. Also Tavakoli Ghouchani et al. (2021) showed that Lack of family support and lack of self-efficacy were the major reasons for abandoning addiction treatment [36]. In this regard, introducing stronger support services in medical protocols can reduce sense of distrust to the extent possible.

Several studies have mentioned concerns about the treatment process and lack of knowledge about priorities for post-treatment and lack of sufficient awareness about selecting the right treatment among the factors for avoiding treatment [18, 37]. Stone (2015) stated that participants in their study were concerned if they started to take methadone for treatment they would never be able to quit [37]. As results of the current study show, too, lack of awareness about treatment is one of the obstacles for considering quitting. Lack of information and lack of exact information about the treatment process causes that individuals feel a sense of confusion, distrust, and even vulnerability due to facing more issues upon considering quitting processes. If these individuals are not properly guided by treatment experts and if they are not provided with correct information, this issue can be a cultural obstacle for quitting. Educating people about chronic nature of substance use and the processes of treatment can improve individuals’ understanding about addiction and treatment, which can cause higher participation in receiving treatment [22].

Results of a review study showed four major obstacles in quitting methamphetamine as: first, psycho-social obstacles which cause shame and stigma; second, lack of belief for necessity of treatment, which rises from a lack of understanding about the used substance and the need for treatment; third, due to unawareness about treatment processes and lack of trust in the effectiveness and efficiency of the services offered by substance dependent treatment centres, some individuals prefer to quit on their own and without any help; and four, there was protecting their personal privacy, which can cause both a fear for exposing one’s personal information, rising from a fear of social stigma and being judged by the staff of treatment centres and others. In fact, fear of social stigma, as shown in the current study and some other studies in this field, can be a social obstacle for quitting substance use [26–28].

In Stone’s study (2015), emphasized the negative attitude of the staff as a significant obstacle for quitting addiction. Results of a review study confirmed negative attitude of medical staff and specialists towards person who uses substance, which can cause violence, influence, and low motivation of the staff for offering services to these patients [37]. On one hand, considering staff’s negative attitude as a stigma can cause doubt towards receiving sufficient medical services and all that is needed, which can reduce the sense of ability and positive results of the treatment and can be a social obstacle for considering treatment. Ashford et al. (2018) interviewed experts in the field to identify systemic obstacles for quitting. They reported that in order to reduce stigma as an obstacle from within the system, it is necessary to provide more education to reduce biased and discriminating beliefs among specialists in the field. These trainings must also be at a larger scale in the society, for example through mass communication [35]. We used a convenient sampling of people that were already in treatment. These individuals were able to overcome any barriers to enter the treatment process. We excluded the people who had not overcome these barriers and had not got to treatment. These two groups of people may be different and may have different obstacles. It is difficult to find people who have not yet overcome barriers to start treatment. Therefore, our sample is a limitation of the present study.

## Conclusion

Findings from this study show that various personal/family, and social factors can serve as barriers for quitting substance use. Findings show that the doubt and inefficiency which included lack of family support and lack of certainty in the individual’s ability for quitting, were one of the obstacles for quitting. Also, social stigma and fear and lack of awareness about treatment were other obstacle.

It is also necessary to emphasise and provide training in terms of expectations about pain management, symptoms of quitting, methods and approaches for working with doctors and nurses to facilitate treatment processes. It seems that better communication among staff and specialists in this field with patients can help reduce sense of confusion, social stigma, and thereby improve treatment. Also, considering lack of family support as an obstacle for quitting, it is necessary to educate these individuals’ families how to provide support so that with the right support the person who uses substance’ attempt for quitting can be facilitated.

Therefore, considering the necessity of using a validated tool for planning and evaluating effective interventions for substance users is helpful. The Substance Abuse Treatment Barriers Questionnaire is designed with 10 items and 3 dimensions, which has appropriate validity and reliability and can be used to determine the obstacles for treatment or factors that lead to discontinuing treatment.

## Data Availability

Not applicable.

## Abbreviation

CVR: Content Validity Ratio,
CVI: Content Validity Index
MMT: Methadone maintenance treatment
SUTBQ: Substance Use Treatment Barriers Questionnaire
